# A toolkit of tagged *glp-1* alleles reveals strong *glp-1* expression in the germline, embryo, and spermatheca

**DOI:** 10.17912/micropub.biology.000271

**Published:** 2020-06-22

**Authors:** Erika B Sorensen, Hannah S Seidel, Sarah L Crittenden, Joseph H Ballard, Judith Kimble

**Affiliations:** 1 Wabash College, Crawfordsville, IN; 2 Eastern Michigan University, Ypsilanti, MI; 3 University of Wisconsin-Madison and HHMI, Madison, WI

**Figure 1:**
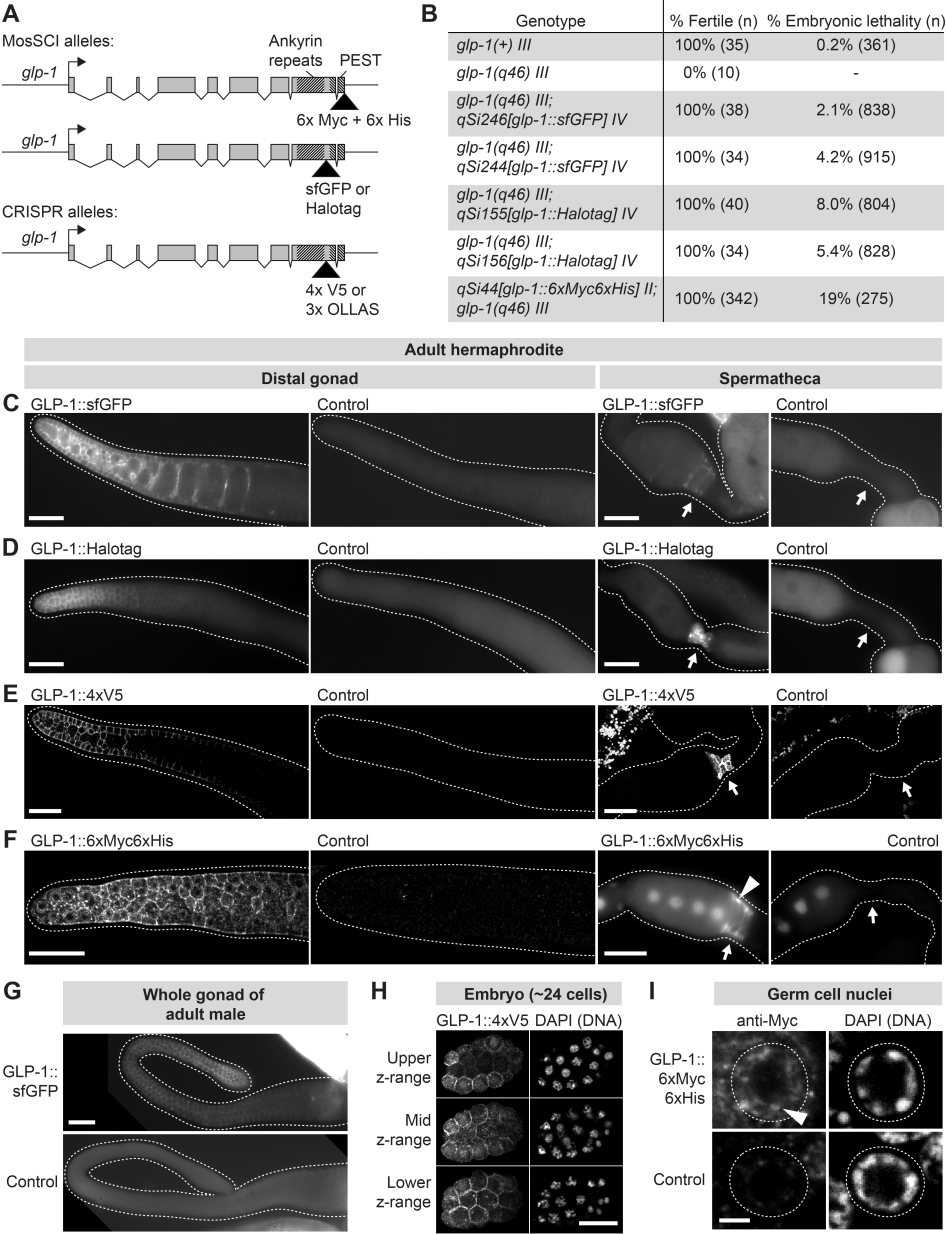
Tagged *glp-1* alleles are expressed in the distal germline, embryo, and spermatheca. (A) Schematic of tagged *glp-1* alleles. (B) Rescue of *glp-1* null phenotype by MosSCI *glp-1* alleles. (C-F) Adult hermaphrodite gonads, oriented distal to the left. Arrow, spermatheca. (F) Arrowhead, GLP-1::6xMyc6xHis visible within spermathecal cell nucleus. Staining in oocyte nuclei likely includes a large component of background staining because staining is present at similar levels in controls. (G) Adult male gonads. (C-G) Dashed line, outline of gonad. (H) Embryo expressing GLP-1::4xV5. Images are maximum-intensity z-projections through equal z-ranges of the same embryo. Anti-V5 staining in control embryos (not shown) was uniformly dim. (C-H) Scale bar, 20 µm. (I) Distal-most germ cell nuclei of adult hermaphrodite. Dashed line, germ cell nucleus. Arrowhead, GLP-1::6xMyc6xHis visible within germ cell nucleus. Scale bar, 2 µm. (C-G, I) Control, animals lacking a tagged *glp-1* allele.

## Description

The *C. elegans* genome encodes two Notch receptors, GLP-1 and LIN-12 (Greenwald and Kovall, 2013). These receptors function in multiple tissues to regulate development, behavior, and reproduction. GLP-1 controls cell fate decisions in the germline (Kimble and Seidel, 2013) and early embryo (Priess, 2005). LIN-12 controls development of the vulva (Greenwald, 2005; Sternberg, 2005). These receptors also act outside the context of development in post-mitotic neurons to regulate chemosensation (Singh *et al.*, 2011), locomotion (Chao *et al.*, 2005), and life-history decisions (Ouellet *et al.*, 2008). Additional processes regulated by Notch signaling include oocyte growth (Nadarajan *et al.*, 2009) and ovulation (McGovern *et al.*, 2018). Understanding the many functions of Notch signaling therefore requires a global picture of Notch receptor expression.

Previous efforts to visualize Notch receptor expression have focused on *lin-12* expression in the soma and *glp-1* expression in the germline and early embryo. LIN-12 has been visualized successfully using LIN-12::GFP fusion proteins introduced via traditional, multi-copy transgenic techniques (e.g. Levitan and Greenwald, 1998; Sarov *et al.*, 2012). Such techniques have been difficult for visualizing *glp-1* expression in the germline, until recently (Cinquin *et al.*, 2015; Gutnik *et al.*, 2018), due to germline silencing of multi-copy transgenes (Kelly *et al.*, 1997; Merritt and Seydoux, 2010). Visualizing GLP-1expression in the germline has therefore relied on antibody staining, primarily using an antibody raised against the GLP-1 extra-cellular domain (Crittenden *et al.*, 1994). This antibody reveals easily detectable GLP-1 protein at the plasma membrane in the distal germline and early embryos (Crittenden *et al.*, 1994; Evans *et al.*, 1994), but it does not allow visualization of the GLP-1 nuclear intracellular domain (NICD), which moves into the nucleus upon receptor activation. This antibody is therefore not useful for identifying cells that have directly received active GLP-1 signaling. To overcome this limitation, and to broaden the toolkit for visualizing *glp-1* expression, we created five strains expressing tagged *glp-1* alleles, including transgenes and CRISPR tags at the endogenous locus. We report here the expression patterns of the tagged *glp-1* alleles, focusing on the adult gonad.

We created five tagged *glp-1* alleles, each expressing GLP-1 protein tagged with one of the following tags: sfGFP (~27 kDa), Halotag (~33 kDa), 4xV5 (~7 kDa), 3xOLLAS (~4.4 kDa), or 6xMyc6xHis (~8 kDa). The 6xMyc6xHis tag was placed at the C-terminus of *glp-1*, downstream of the GLP-1 PEST domain (Figure 1A). The remaining tags were placed C-terminal to the GLP-1 Ankyrin repeats but upstream of the PEST domain (Figure 1A). These sites place the tags at or near the C-terminus of the NICD. Strains expressing *glp-1::sfGFP*, *glp-1::Halotag*, and *glp-1::6xMyc6xHis* were created via Mos1-mediated single-copy insertion of the transgene into the genome. Strains expressing *glp-1::4xV5* and *glp-1::3xOLLAS* were created by CRISPR-mediated insertion of the tag into the endogenous *glp-1* locus.

To assess functionality of the tagged GLP-1 proteins, we tested each for rescue of the *glp-1* loss-of-function phenotype, which includes infertility and maternal-effect embryonic lethality. Rescue by *glp-1::sfGFP*, *glp-1::Halotag*, and *glp-1::6xMyc6xHis* was assessed by crossing each transgene into animals carrying the null allele *glp-1(q46)* (Kodoyianni *et al.*, 1992). Rescue by the CRISPR-generated alleles (*glp-1::4xV5* and *glp-1::3xOLLAS*)was assessed by examining animals homozygous for the tagged allele. We observed strong rescue for all tagged *glp-1* alleles: Animals expressing *glp-1::4xV5* or *glp-1::*3xOLLAS were all fertile (n > 50 hermaphrodites per allele) and showed no noticeable embryonic lethality; animals expressing *glp-1::6xMyc6xHis,* *glp-1::sfGFP* or *glp-1::Halotag* were all fertile, but showed a low penetrance embryonic lethality (Figure 1B). We conclude that all five tagged *glp-1* alleles encode functional GLP-1 proteins.

We characterized expression of the tagged *glp-1* alleles in adult hermaphrodites and males, focusing on the gonad. GLP-1::6xMyc6xHis, GLP-1::4xV5, and GLP-1::3xOLLAS were visualized with immunostaining of extruded gonads. GLP-1::sfGFP and GLP-1::Halotagwere examined in live animals and extruded gonads. All five tagged alleles showed expression in two areas: the distal germline and spermatheca (Figure 1C-G). Signal in the spermatheca was strongest at the membrane but also sometimes visible at a lower level in spermathecal nuclei (Figure 1F). Expression in the distal germline was present in both sexes and strongest in the distal-most ~20 rows of germ cells, becoming weaker more proximally (Figure 1C-G). Germ cells in the distal-most gonad showed membrane staining similar to that seen with antibodies to the extracellular domain of GLP-1 (e.g. Crittenden *et al.*, 1994); in addition, staining in the nucleus was detected, but at a low level compared to membrane staining (Figure 1I). This difference in membrane and intranuclear GLP-1 staining is consistent with a previous report of GLP-1 NICD expression in the distal germline (Gutnik *et al.*, 2018). We also examined pre-gastrulation embryos and observed both membrane and nuclear expression of tagged GLP-1, in a pattern consistent with GLP-1 expression in the AB cell lineage (Figure 1H), as reported previously (Evans *et al.*, 1994; Priess, 2005). Expression in L4 and adult animals was not observed outside the gonad or in the male somatic gonad (although we cannot exclude the possibility of expression below our threshold of detection). Spatial patterns of expression were essentially the same for all five tagged *glp-1* alleles, although signal-to-background ratios differed: 4xV5 and Halotag gave strong signal in animals with the tagged *glp-1* alleles and minimal background in controls; sfGFP gave a dimmer signal than 4xV5 and Halotag; and 3xOLLAS and 6xMyc6xHis had higher background in controls, including in germ cell nuclei (data not shown for 3xOLLAS). These results show that the sites of strongest *glp-1* expression in adults are the distal germline and the spermatheca.

Our study provides a toolkit of five tagged *glp-1* alleles. These alleles confirm the expected pattern of GLP-1 expression in the distal germline (Crittenden *et al.*, 1994), and they further enable visualization of the GLP-1 NICD in germ cell nuclei (Figure 1I). These alleles also show strong expression in an unexpected site: the spermatheca (Figure 1C-F). GLP-1 has not been reported previously in the spermatheca. Yet Notch signaling has been proposed to affect passage of the oocyte through the spermatheca because mutations in the Notch ligand *apx-1* cause defects in ovulation (McGovern *et al.*, 2018). In addition, the DNA-binding co-factor of Notch, LAG-1, is strongly expressed in spermathecal nuclei (Chen *et al.*, 2020). Notch signaling in the spermatheca was proposed to be mediated by LIN-12 (McGovern *et al.*, 2018), but our study suggests that GLP-1 is another reasonable candidate. We expect that our toolkit of tagged *glp-1* alleles will prove useful in visualizing GLP-1 in the germ cells, as well as in investigating a possible role for GLP-1 in the spermatheca.

## Methods

**Methods**

**Strains and growth conditions**

Worms were grown at 20°C on standard nematode growth media plates seeded with *E. coli* OP50.

N2

EG8081 *unc-119(ed3) III; oxTi177[ttTi5605 + NeoR(+) + unc-18(+) ] IV*

EG6699 *ttTi5605 II; unc-119(ed3) III; oxEx1578*

JK4862 *glp-1(q46)* III / *hT2[bli-4(e937) let-?(q782) qIs48]* (I;III)

JK5008 *qSi44[Cbr-unc-119 + glp-1::6xMyc6xHis]* II ; *glp-1(q46)* III

JK5525 *glp-1(q46)* III; *qSi155[Cbr-unc-119 + glp-1::Halotag]* IV

JK5526 *glp-1(q46)* III; *qSi156[Cbr-unc-119 + glp-1::Halotag]* IV

JK5535 *glp-1(q46)* III; *qSi246[Cbr-unc-119 + glp-1::sfGFP]* IV

JK5548 *glp-1(q46)* III; *qSi244[Cbr-unc-119 + glp-1::sfGFP]* IV

JK5973 *glp-1(q997[glp-1::3xOLLAS])* III

JK5933 *glp-1(q1000[glp-1::4xV5])* III

**Creation of *glp-1::sfGFP*, *glp-1::Halotag,* and *glp-1::6xMyc6xHis* constructs**

*glp-1::sfGFP* and *glp-1::Halotag* were constructed by cloning the genomic *glp-1* locus into MosSCI vector pCFJ151 (Frøkjaer-Jensen *et al.*, 2008), using Gibson assembly (Gibson, 2011). This construct contains 2,506 bp upstream of the *glp-1* start codon and 934 bp downstream of the *glp-1* stop codon. sfGFP (Pédelacq *et al.*, 2006) and Halotag (Los *et al.*, 2008) were amplified from sfGFP expression plasmid (Sandia Biotech #23004006) and pFN18A (Promega #G2751), respectively, using primers containing *Nru*I sites in primer tails. sfGFP and Halotag were inserted into *glp-1* at the *Nru*I site using standard ligation. *glp-1::6xMyc6xHis* was constructed by cloning the same genomic *glp-1* locus into MosSCI vector pCFJ151, using Gibson assembly, except that unique *Not*I and *Pme*I sites were inserted before the stop codon using primers containing *Not*I and *Pme*I sites in primer tails. The 6xMyc6xHis tag was amplified from plasmid 4myc-deltaE pAdtet7 (Sorensen and Conner, 2010) and inserted between the *Not*I and *Pme*I sites using standard ligation.

**Single-copy insertion of *glp-1::sfGFP*, *glp-1::Halotag*, and *glp-1::6xMyc6xHis***

*glp-1::sfGFP* and *glp-1::Halotag* were integrated into site *oxTi177* on LGIV by injection of their respective constructs into strain EG8081 using the Universal MosSCI technique, as described (Frøkjær-Jensen *et al.*, 2014). *glp-1::6xMyc6xHis* was integrated into site *ttTi5605* site on *LGII* by injection of the *glp-1::6xMyc6xHis* construct into strain EG6699, as described (Frøkjær-Jensen *et al.*, 2012). Injection mix contained 15 ng/µl *glp-1::sfGFP* or *glp-1::Halotag* or *glp-1::6xMyc6xHis*, 50 ng/µl pCFJ601, 10 ng/µl pGH8, 25 ng/µl pCFJ90, 5 ng/µl pCFJ104, and sometimes 10 ng/µl pMA122. Positive insertions were recovered based on the Non-Unc phenotype and loss of co-injection markers. Insertions were crossed into a *glp-1(q46)* genetic background. Homozygosity of the *glp-1(q46)* allele was confirmed using primers that amplify only the endogenous locus of *glp-1* (outer forward, 5’-aaacactttttgggtgctgtg-3’; inner forward, 5’-gatttgaactgccatgatttat-3’; inner reverse, 5’-acagcttgccgatacctgc-3’; outer reverse, 5’-tcagttcattgatcttgtcgacac-3’) followed by digestion with *Mse*I.

**CRISPR/Cas9 genome editing to create *glp-1::4xV5* and *glp-1::3xOLLAS***

*glp-1::4xV5* and *glp-1::3xOLLAS* were generated via a co-CRISPR genome editing strategy using a CRISPR/Cas9 RNA-protein complex (Arribere *et al.*, 2014; Paix *et al.*, 2015). Wildtype animals were injected with a mix containing a gene-specific crRNA (10 μM, IDT-Alt-R^TM^), *unc-58* co-CRISPR crRNA (4 μM, IDT-Alt-R^TM^), tracrRNA (13.6 μM, IDT-Alt-R^TM^), gene specific repair oligo (4 μM), *unc-58* repair oligo (1.34 μM), and Cas-9 protein (24.5 μM). F1 progeny of injected hermaphrodites were screened for desired mutations by PCR and Sanger sequencing. Each allele was outcrossed against wild-type twice prior to analysis. The *glp-1*-specific crRNA was 5’-CAG GUC AUG GUG CUA AGU CUG UUU UAG AGC UAU GCU-3’. The repair oligo for *glp-1::4xV5* was 5’-GCT CTT TTG ATA TTC TTC ACA GTT TGT CGC CCA GAG GTG CTA TCA AGT CCG AGG AGT GGG TTA GGG ATA GGC TTA CCG GTG CTA TCA AGT CCG AGG AGT GGG TTT GGG ATT GGC TTT CCA GTA CTA TCT AGA CCG AGG AGA GGG TTA GGG ATA GGC TTA CCC TTA GCA CCA TGA CCT GAC TTG ACT ATT TGC TG-3’. The repair oligo for *glp-1::4xV5* contained three copies of the V5 tag, but the insertion recovered contained four copies of the V5 tag. The repair oligo for *glp-1::3xOLLAS* was 3’-GCT CTT TTG ATA TTC TTC ACA GTT TGT CGT CCA GAC TTT CCC ATG AGA CGT GGT CCG AGC TCG TTG GCG AAT CCG GAT TGC TTT CCC ATG AGA CGT GGT CCG AGC TCG TTG GCG AAT CCG GAT TGC TTT CCC ATG AGA CGT GGT CCG AGC TCG TTG GCG AAT CCG GAC TTA GCA CCA TGA CCT GAC TTG ACT ATT TGC TG-3’. Primers used to genotype animals for insertions were SLR10 (5’-AACTCTGTGGGGACCAGTG-3’) and SLR21 (5’-GATGCTAGCTGTTCGTGC-3’).

**Locations of tags within the GLP-1 protein sequence**

The 4xV5 and 3xOLLAS tags were inserted between lysine 1185 and serine 1186 (…SGHGAK-tag-SGRQTV…). sfGFP and Halotag were inserted between serine 1209 and arginine 1210 (…TSAASS-tag-RETNHL…). The 6xMyc6xHis tag was inserted immediately upstream of the stop codon (…NGSFYC-tag-*).

**Rescue of *glp-1(q46)***

Fertility was assessed by examining hermaphrodites aged 24-hrs post mid-L4 at 400X magnification. Animals were scored as ‘fertile’ if embryos were present in both sides of the uterus and if both arms of the gonad were similar in size and morphology to those of wildtype animals. Embryo viability was assessed by allowing hermaphrodites aged 24-hrs post mid-L4 to lay eggs for ~8 hrs. Embryos were scored as ‘viable’ if they hatched within ~16 hours after removal of the parent animal from the plate.

**Fixation, staining, and imaging**

Animals were collected as L4s or as adults aged 24 hours post mid-L4. In all experiments, animals expressing tagged *glp-1* alleles were treated in parallel with control animals lacking the allele.

For *glp-1::4xV5* and *glp-1::3xOLLAS*, gonads were extruded in 0.25 mM levamisole/PBSTw (PBS + 0.1% Tween-20) and fixed in 4% (w/v) paraformaldehyde/PBSTw for 10 min at room temperature. Gonads were permeabilized in 0.1%Triton X-100/PBSTw for 30 min and blocked in PBSB (PBSTw + 0.5% BSA) for 20 min at room temperature. Gonads were stained overnight at 4°C in mouse anti-V5 (Bio-Rad #MCA1360) or rat anti-OLLAS(Novus #NBP1-06713) diluted 1:1000 in block. Gonads were washed three times in block for 10 min per wash and incubated for 1 hour at room temperature in 0.1 µg/ml DAPI and AlexaFluor 555 donkey anti-mouse (Invitrogen #A31570), AlexaFluor 647 donkey anti-mouse (Invitrogen #A31571), or AlexaFluor 488 donkey anti-rat (Invitrogen #A21208) diluted 1:1000 in block. Gonads were washed as before and mounted in ProLong Gold (Thermo-Fisher #P36934). For staining of embryos, gravid hermaphrodites were cut on a subbed slide in PBS to release embryos. Paraformaldehyde was added to 4% (w/v), and embryos were depressed under a coverslip to crack the eggshell. The slide was incubated in a moist chamber for 30 min at room temperature and frozen on dry ice for 5-10 min. The coverslip was removed, and the slide was immersed in -20ºC methanol for 10 min, followed by room temperature methanol for 10 min. Embryos were blocked and stained with antibodies as described for gonads.

For *glp-1::6xMyc6xHis*, gonads were extruded in 0.2 mM levamisole/PBSTw and fixed in 3% (w/v) paraformaldehyde/0.1 M K_2_HPO_4_ (pH 7.2) for 20 min at room temperature. Gonads were washed once in PBSTw and permeabilized in -20°C methanol for 5 min. Gonads were washed three times in PBSTw and blocked in PBSB for 45 min at room temperature. Gonads were stained overnight at 4°C in mouse monoclonal antibody 9E10 diluted 1:10 in block. The 9E10 antibody, developed by Michael J. Bishop, was obtained from the Developmental Studies Hybridoma Bank, created by the NICHD of the NIH (The University of Iowa, Department of Biology, Iowa City, IA 52242). Gonads were washed four times in PBSTw for 15 min total and incubated for 1 hour at room temperature in 0.1 µg/ml DAPI and AlexaFluor 555 goat anti-mouse (Thermo-Fisher #A28180) diluted 1:1000 in block. Gonads were washed as before and mounded in Vectashield (Vectorlabs #H-1000).

For *glp-1::sfGFP*, whole living animals were imaged on agarose pads. For imaging of gonads, gonads were extruded, fixed, and permeabilized as for *glp-1::6xMyc6xHis*. Gonads were washed three times in PBSTw and mounted in Vectashield.

For *glp-1::Halotag*, animals were incubated for 90 min in HaloTag® TMR Ligand (Promega, #G8252) diluted 1:1000 in M9/0.1% Tween-20. Animals were rinsed once with M9/0.1% Tween-20 and allowed to crawl around for 30 min on a seeded plate in the dark. For imaging of whole animals, live animals were mounted on agarose pads and imaged immediately. For imaging of gonads, gonads were extruded in 0.2 mM levamisole/PBSTw and fixed in 3% (w/v) paraformaldehyde/0.1 M K_2_HPO_4_ (pH 7.2) for 20 min at room temperature. Gonads were mounted in Vectashield. For *glp-1::Halotag* embryos (images not shown), gravid hermaphrodites were cut on a subbed slide in PBS/100 nM HaloTag® TMR Ligand to release embryos. Paraformaldehyde was added to 4% (w/v), and embryos were incubated for 10 min in a moist chamber. Embryos were depressed under a coverslip to crack the eggshell, and the slide was frozen on dry ice for 5-10 min. The coverslip was removed, and the slide was washed in PBSTw. Embryos were mounted in ProLong Gold.

Images of *glp-1::sfGFP*, *glp-1::Halotag*, and *glp-1::6xMyc6xHis* (spermatheca) were acquired on a Zeiss AxioScope A1 microscope equipped with a AxioCam 503 mono camera. Images of *glp-1::4xV5* and *glp-1::6xMyc6xHis* (distal germline) were acquired on a Leica SP8 laser scanning confocal microscope. For each set of images, brightness levels were adjusted the same for experimental and control images.
