# Corrigendum: A toolkit of tagged glp-1 alleles reveals strong glp-1 expression in the germline, embryo, and spermatheca

**DOI:** 10.17912/micropub.biology.000290

**Published:** 2020-08-11

## Description

For Sorensen, EB; Seidel, HS; Crittenden, SL; Ballard, JH; Kimble, J (2020). A toolkit of tagged glp-1 alleles reveals strong glp-1 expression in the germline, embryo, and spermatheca. microPublication Biology. 10.17912/micropub.biology.000271.

The authors correct the following:

In the Methods section: The strain ‘**JK5525** glp-1(q46) III; qSi155[Cbr-unc-119 + glp-1::Halotag] IV’ is corrected to ‘**JK5225** glp-1(q46) III; qSi155[Cbr-unc-119 + glp-1::Halotag] IV’

The strain ‘**JK5526** glp-1(q46) III; qSi156[Cbr-unc-119 + glp-1::Halotag] IV’ is corrected to ‘**JK5226** glp-1(q46) III; qSi156[Cbr-unc-119 + glp-1::Halotag] IV’

Throughout the text and in the Methods section:

The genotype of strain JK5973 “[glp-1::**3**xOLLAS]” is corrected to: “[glp-1::**2**xOLLAS]”

